# The impacts of medication shortages on patient outcomes: A scoping review

**DOI:** 10.1371/journal.pone.0215837

**Published:** 2019-05-03

**Authors:** Jonathan Minh Phuong, Jonathan Penm, Betty Chaar, Lachlan Daniel Oldfield, Rebekah Moles

**Affiliations:** Sydney Pharmacy School, Faculty of Medicine and Health, University of Sydney, Sydney, Australia; Leiden University Medical Center, NETHERLANDS

## Abstract

**Background:**

In recent years, medication shortages have become a growing worldwide issue. This scoping review aimed to systematically synthesise the literature to report on the economic, clinical, and humanistic impacts of medication shortages on patient outcomes.

**Methods:**

Medline, Embase, Global Health, PsycINFO and International Pharmaceutical Abstracts were searched using the two key concepts of medicine shortage and patient outcomes. Articles were limited to the English language, human studies and there were no limits to the year of publication. Manuscripts included contained information regarding the shortage of a scheduled medication and had gathered data regarding the economic, clinical, and/or humanistic outcomes of drug shortages on human patients.

**Findings:**

We found that drug shortages were predominantly reported to have adverse economic, clinical and humanistic outcomes to patients. Patients were more commonly reported to have increased out of pocket costs, rates of drug errors, adverse events, mortality, and complaints during times of shortage. There were also reports of equivalent and improved patient outcomes in some cases.

**Conclusions:**

The results of this review provide valuable insights into the impact drug shortages have on patient outcomes. The majority of studies reported medication shortages resulted in negative patient clinical, economic and humanistic outcomes.

## Introduction

Medicines are an essential part of medical care, which improve patients' health and quality of life [1]. In the modern era, with the advancement of manufacturing, distribution, and transport technologies, one would expect medication shortages to be a minor issue, where supply is quickly resolved without negative ramifications to patient health and quality of life. Unfortunately, this is not the case. Medication shortages have been described as a growing worldwide issue in recent years [2].

There are various definitions of medication shortages, often also called “drug shortages”. The University of Utah Drug Information Service defines a shortage as "A supply issue that affects how pharmacies prepare and dispense a product or that influences patient care when prescribers must choose an alternative therapy because of supply issues” [3]. The US Food and Drug Administration (FDA) defines medication shortages as "a period of time when the demand or projected demand for the drug … exceeds the supply of the drug" [3]. Despite minor differences in definitions, if a medicine is not available for the patient at the time they require treatment, regardless of the reason, the patient will either have to go without treatment, choose an alternative treatment, delay treatment, or incur some difficulty by trying to obtain treatment via another source.

Multiple reasons have been cited as to why drug shortages occur. These include quality manufacturing issues, insufficient quantities of raw materials, regulatory issues, discontinuation of products from the market, procurement issues, business decisions and natural disasters [4–7]. In order to overcome the impact of shortages on the health system and patients, various strategies have been implemented such as changes to policies, increased reporting and expedited approval [8]. In fact, some of these strategies have worked to a small extent as the FDA reported a decrease in drug shortages in recent years from 117 in 2012 to 23 in 2016 [6]. Despite these efforts, at the time of writing this manuscript, there were 91 medications listed to be in shortage on the FDA website, [9] and the University of Utah Drug Information Services showed a stable trend of around 150 shortages each year [10]. In Australia, a snapshot by the Society of Hospital Pharmacists Australia reported 365 different medication shortages on a single day in 2016 [11]. These numbers indicate just how complex this global problem is and that further work needs to be done to ensure access to essential medicines when they are required.

As alluded to, the ramifications of medication shortages have vast implications for both the health care system, health institutions and workforce. Importantly, medication shortages have the potential to impact patient outcomes. The literature has reported that shortages have put economic burdens on the health system, interfered with patient care, and have led to patient dissatisfaction [12, 13]. Medication shortages have also been reported to increase patient out of pocket costs [13, 14] and most alarmingly they have led to adverse patient events including mortality, treatment changes, inferior treatment, and medication errors [13, 15]. Some positive or equivalent outcomes have emerged from using alternative treatments as workarounds [16]. Despite these reports, there have been no reviews exploring the economic, clinical and humanistic impacts of medication shortages on patient outcomes. Therefore, this scoping review aimed to systematically synthesise the literature to report on the economic, clinical, and humanistic impacts of medication shortages on patient outcomes.

## Methods

### Search

Utilising the Preferred Reporting Items for Systematic Reviews and Meta-Analyses (PRISMA) protocol [17], this scoping review gathered research manuscripts that reported patient outcomes related to medicines shortages (S1 File). Medline, Embase, Global Health, PsycINFO and International Pharmaceutical Abstracts were searched using the two key concepts of medication shortage and patient outcomes (S2 File). Searches were limited to the English language, human studies and there were no limits to the year of publication.

### Study selection

After the removal of duplicates, conference abstracts, editorials, and opinion pieces, titles and abstracts were scanned for relevance. Manuscripts were included if they were about a shortage of a scheduled medicine, had gathered data regarding the economic, clinical, and/or humanistic outcomes of drug shortages on human patients, and followed the IMRAD format [18] (containing an introduction, methods, results and discussion section). Manuscripts were excluded if the impact to patients was not attributed to shortages.

### Data extraction

Study location, data collection method, the medicines(s) affected by the shortage, and patient outcomes were extracted from each manuscript. Economic outcomes were those that directly affected a patients’ finances as a consequence of the shortage; clinical outcomes were defined as a change in a patient's health status due to a drug shortage. Subsets of clinical outcomes included mortality; adverse drug reactions; drug errors; changes in hospitalisation, such as increased length of stay or readmission; and other clinical outcomes, e.g. changes in a measured parameter such as blood pressure. Humanistic outcomes were defined as non-clinical and non-economic impacts on patients such as quality of life, satisfaction and concerns as a result of the shortage.

## Results

**Fig 1:**
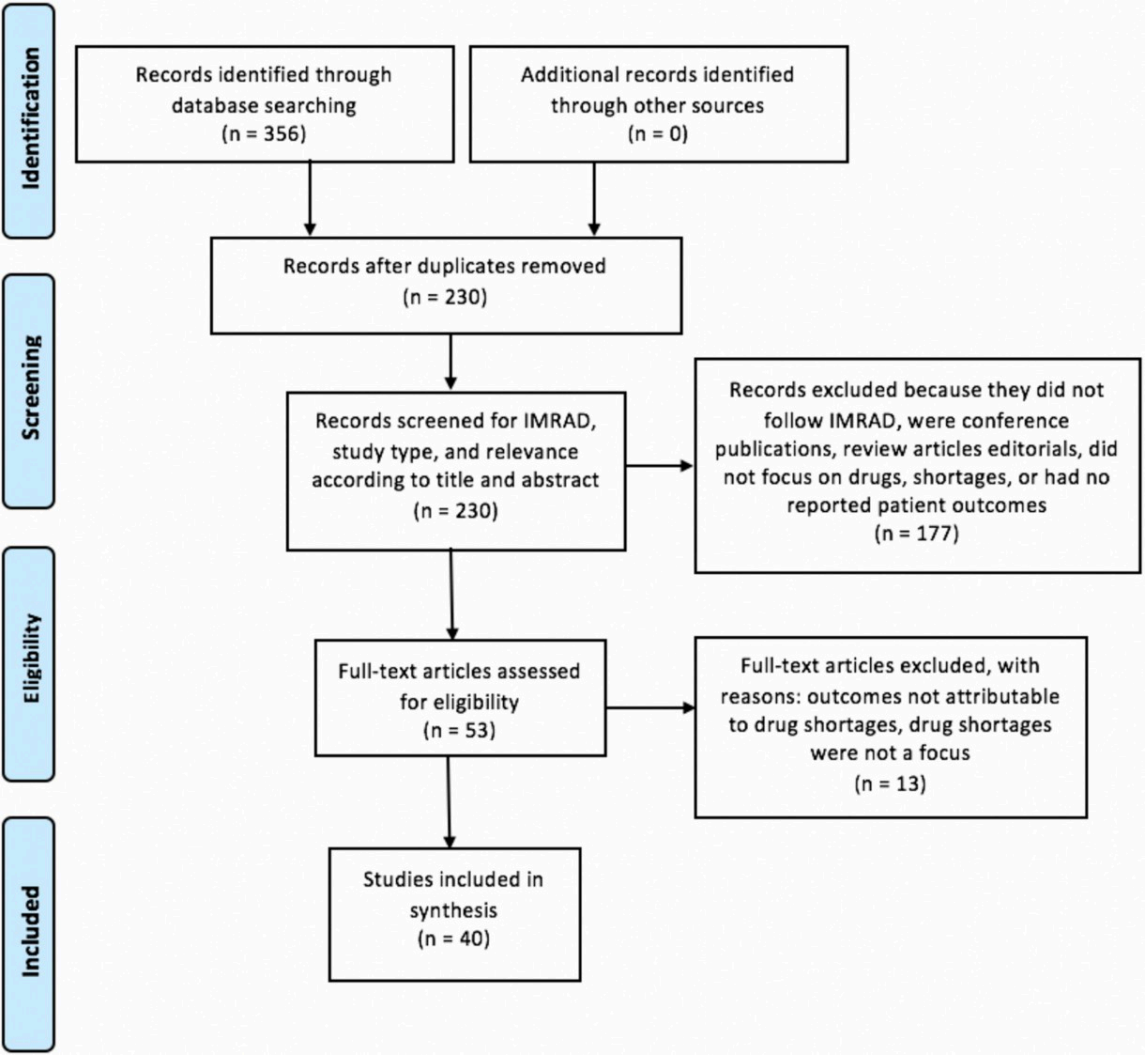
PRISMA flowchart.

A total of 230 unique articles were found after the initial search. After application of inclusion and exclusion criteria, 40 manuscripts were included in the final review (Fig 1). These 40 studies all contained data regarding economic, clinical, and/or humanistic impacts of drug shortages on patients.

Included manuscripts were from 11 countries worldwide. The majority of manuscripts were from North America (n = 28), followed by Africa (n = 5), Europe (n = 3) and the Western Pacific (n = 3) the remaining manuscript was from Saudi Arabia in the Middle East. All manuscripts were observational studies, retrospective cohort studies (n = 19) and surveys (n = 15). Studies gathered information from patient charts (n = 21), reports of healthcare professionals (n = 19), and patient reports (n = 3). Some studies used multimodal methods to gather data on patient outcomes due to shortages.

There were 19 studies which reported on an individual medicine, of these 15 were unique medicines. Three medicines were reported multiple times: daunorubicin (n = 2), piperacillin-tazobactam (n = 2) and propofol (n = 3). In 13 studies, a group of medicines was reported, of these, there were seven unique groups. The most common groupings were: oncology medicines (n = 6), antimicrobials (n = 2). Other groups were anaesthetics, antihypertensives, antiretrovirals, paediatric medicines and glaucoma medicines. Eight studies did not specify a particular medicine in shortage, instead they reported on medication shortages in general.

### Economic outcomes

Economic outcomes due to medication shortages were reported in five studies (Table 1). The only economic outcome reported was patient out of pocket (OOP) costs. All five studies reported an increase in patient OOP costs [7, 19–22].

**Table 1:** Economic outcomes of drug shortages on patients.

First author and year	Location	Study Description	Shortage medicine	Type of outcome	Description of outcome
Pauwels, 2015 [19]	Europe	Survey of hospital pharmacists on the characteristics, clinical impact, financial impact, and management of drug shortages in European hospital pharmacies.	General medicines	Out of pocket costs	Increased patient OOP costs were reported to impact patients: always-often (11%), sometimes (13%), rarely-never (45%), no answer (31%).
Tan, 2016 [7]	Australia	Semi-structured interviews of community pharmacists on the causes, impact and management strategies of medication shortages in the community setting.	General medicines	Out of pocket costs	Pharmacists reported that patients had increased expenses trying to acquire medications in shortage.
Perumal-Pillay, 2017 [20]	South Africa	Focus groups of parents and guardians of children to gather opinions and perceptions on the availability of children's medications.	Paediatric medicines	Out of pocket costs	Free medicines were not always available at government facilities, so participants were sometimes forced to pay for medications at private sector pharmacies.
Hayes, 2014 [21]	USA	Retrospective cohort study using data from patient records. Leucovorin shortage resulted in levoleucovorin use.	Leucovorin	Out of pocket costs	The mean annual patient OOP costs were $167 to $714 higher for levoleucovorin than for leucovorin.
Lukmanji, 2017 [22]	Canada	Mixed methods longitudinal cohort study using open-ended surveys of patients and patient records to study the impact of a clobazam shortage on patients with epilepsy.	Clobazam	Out of pocket costs	Patients reported having to pay up front for medication without receiving the full prescription and increased gas costs from having to drive further distances to find supply.

OOP = Out of pocket

### Clinical outcomes

Clinical outcomes due to medication shortages were reported in 38 studies (Table 2). Mortality was reported in 16 studies. Of these, ten reported increased mortality [5, 13, 23–30], and five reported equivalent mortality [31–35]. One study, Abdelrahman et al. [36] reported both increased and equivalent mortality, the majority of physicians agreed or were neutral regarding the statement that drug shortages resulted in patient deaths, however, there was also a proportion of physicians which disagreed. Medication classes that were commonly involved in poor patient outcomes during shortages included antimicrobials [28, 33, 37–39] and oncology medicines [24–27, 31, 32, 40–42].

**Table 2:** Clinical outcomes of drug shortages on patients.

First author and year	Location	Study Description	Shortage	Type of outcome	Description of outcome
Abdelrahman, 2016 [36]	Egypt	Survey of physicians on perceived health outcomes of drug shortages on patients.	General medicines	Mortality ADR Drug error Other	Death as a result of drug shortage was reported by 67 physicians (35%). Most physicians agreed or were neutral with regards to statements that drug shortages: add to patient suffering and may cause condition deterioration; delay surgical operations; increase patient suffering from illness without improvement; result in patients receiving wrong medicines; result in patients’ condition deteriorating; physicians having to treat via a surgical operation; that analogues and alternative treatments were perceived to cause increased side effects and not give the same effect as the shortage medications.
AlRuthia, 2017 [12]	Saudi Arabia	Questionnaire of pharmacists about drug shortages in hospitals.	General medicines	Other	Eighty-eight percent of hospital pharmacists reported that drug shortages compromise the quality of patient care.
Baumer, 2004 [23]	USA	Survey of directors of pharmacy on the impact of drug shortages in acute care hospitals.	General medicines	Mortality ADR Drug error Other	Pharmacy directors believed that drug shortages had compromised patient care, 65% reported having a procedure delayed or cancelled, 31% reported a prolonged patient stay, 10% reported a serious medication error, 4% reported a serious adverse drug reaction, and 1% reported death.
Caulder, 2015 [46]	USA	Survey of directors of pharmacy on the impact of drug shortages on Health System Pharmacies in the Southeastern United States.	General medicines	Drug error Other	Respondents reported shortages causing drug changes as well as causing a 1–5% error rate and unsafe conditions for patients.
McLaughlin, 2013 [13]	USA	Survey of pharmacy directors assessing the effects of patient care caused by drug shortages.	General medicines	Mortality ADR Drug error Hospitalisation Other	Instances of treatment failure and medication errors were attributed to shortages. Serious adverse events due to shortage were reported. Five reported permanent patient harm, nine instances required intervention to sustain life, and two deaths were reported. Readmission, increased length of stay and treatment delays were also reported.
Pauwels, 2015 [19]	Europe	Survey of hospital pharmacists on the characteristics, clinical impact, financial impact, and management of drug shortages in European hospital pharmacies.	General medicines	Drug error Other	Drug shortages were reported to result in drug errors and inferior treatment.
Tan, 2016 [7]	Australia	Semi-structured interviews of community pharmacists on the causes, impact and management strategies of medication shortages in the community setting.	General medicines	ADR Other	Patients were reported to have adverse events such as withdrawal symptoms due to shortage. Pharmacists noted that the standard workaround is to use a therapeutic equivalent, however, sometimes there is no alternative treatment, causing health consequences.
Walker, 2017 [5]	Fiji	Semi-structured interviews of key stakeholders in the Fijian medicine supply chain.	General medicines	Mortality Hospitalisation Other	Medicine shortages were causing ill-health. Interviewees reported that shortages resulted in longer hospital stays, shorter times to patient readmission and even mortality
Becker, 2013 [40]	USA	Mixed methods study. Retrospective cohort study using data from patient records and survey of treating physicians on the impact of oncology drug shortages on patient therapy.	Oncology medicines	ADR Other	Respondents reported alternative therapy having to be used due to shortage. Alternative agents were reported to have greater toxicity in 34.8% of cases, and less toxicity in 8.9%. The physicians reported that inferior alternative treatments were used in 30.4% of cases.
Duan, 2016 [41]	USA	Prospective study. Alternative vehicles of oncology medications were used in transcatheter arterial chemoembolization following shortages.	Oncology medicines	ADR Other	Similar clinical profiles and toxicity rates were reported between the groups.
Goldsack, 2014 [24]	USA	Survey of pharmacists on the impact of shortages of injectable oncology drugs on patient care.	Oncology medicines	Mortality ADR Drug error Other	Sixty-two percent of respondents reported using alternative drug regimens and 25% reported safety events had occurred in the last 12 months due to drug shortages. Perceived risks were reported such as incorrect doses, adverse drug reactions, and death.
Kehl, 2014 [25]	USA	Survey of oncologists to explore their experiences with drug shortages.	Oncology medicines	Mortality ADR Other	Respondents reported having to use a less effective alternative, delays in patient treatment or other adverse effects on patients. Three oncologists described shortages that they believed contributed to patient deaths.
McBride, 2013 [26]	USA	Survey of oncology pharmacists on the effect of oncology drug shortages on cancer care.	Oncology medicines	Mortality ADR Drug error Other	Survey respondents reported deaths, drug errors, disease progression, and adverse drug effects as a result of drug shortages. Dose reductions were reported in efforts to conserve supply for future doses.
Salazar, 2015 [42]	USA	Survey of pharmacists and principal investigators on the impact of chemotherapy shortages in paediatric oncology.	Oncology medicines	Drug error Other	Two-thirds of principal investigators reported that at least one patient had been impacted by drug shortage. One-third of pharmacists reported at least one near miss or actual errors due to drug shortage.
Berger, 2014 [27]	USA	Retrospective cohort study using data from patient records. Non-FDA-approved second-generation liposomal doxorubicin (Lipo-Dox) was used in recurrent epithelial ovarian carcinoma during a shortage of PEGylated liposomal doxorubicin (Doxil).	PEGylated liposomal doxorubicin (Doxil)	Mortality Other	No patients had a complete or partial response during the treatment period to the alternative therapy. Nine patients died during the study period from their disease.
Nickel, 2014 [31]	USA	Retrospective cohort study using data from patient records. Mitoxantrone was used in induction therapy in newly diagnosed lymphoblastic leukaemia and lymphoma due to daunorubicin shortage.	Daunorubicin	Mortality ADR Hospitalisation Other	Induction toxicities including deaths, ICU admissions, fever, bacteraemia, and invasive fungal disease were similar during shortage. Mean LOS during induction was also similar. Minimal residual disease prevalence at the end of induction was not significantly different.
Trifilio, 2013 [32]	USA	Retrospective cohort study using data from patient records. Idarubicin was used for remission induction in patients with acute myeloid leukaemia (AML) due to shortage of daunorubicin.	Daunorubicin	Mortality ADR Other	Idarubicin was used instead of daunorubicin due to shortage resulting in similar rates of complete remission, all-cause mortality, and adverse drug reactions. Subset analysis revealed patients over 55 years old had significantly higher rates of complete remission using the alternative drug idarubicin.
Gundlapalli, 2013 [28]	USA and Puerto Rico	Survey of infectious disease specialists on their perspectives and concerns regarding antimicrobial shortages.	Antimicrobials	Mortality ADR Hospitalisation Other	Infectious disease specialists expressed an opinion that the resulting change in treatment due to the shortage had adversely affected patient care or outcomes. Of these, the most common concerns were the use of more toxic antimicrobials, broader-spectrum antimicrobials, long-term morbidity from inadequate treatment of infection and longer hospitalisations. Use of suboptimal, less-effective or more toxic therapy was the most commonly mentioned additional adverse outcome in an open-text field. Physicians also reported five deaths attributed directly to antimicrobial shortages.
McLaughlin, 2014 [37]	USA	Survey of clinicians to report patient harms due to antimicrobial shortages.	Antimicrobials	ADR Hospitalisation	Side effects and patient readmission was attributed to shortage due to delayed and unavailable treatment.
Dilworth, 2014 [33]	USA	Retrospective cohort study using data from patient records. Alternative agents were used in patients with HIV *Pneumocystis jirovecii* pneumonia (PJP) instead of trimethoprim-sulfamethoxazole due to shortage.	IV trimethoprim-sulfamethoxazole	Mortality ADR Hospitalisation	Shortage of IV trimethoprim-sulfamethoxazole resulted in alternative agents used for HIV-PJP. Three patients in both groups died. Worsening clinical status was significantly higher in the shortage group. Treatment failure, adverse events, and LOS were recorded however were not significantly different.
Gross, 2018 [38]	USA	Retrospective cohort study using data from patient records. Alternative antimicrobials were used instead of piperacillin-tazobactam.	Piperacillin-tazobactam	ADR	Most hospitals shifted towards the use of high-risk antimicrobials. Of these hospitals which shifted towards high-risk antimicrobials, *Clostridium difficile* infections increased RR = 1.3 (p<0.5).
Mendez, 2006 [39]	USA	Retrospective cohort study using data from patient records. Alternative antimicrobials were used instead of piperacillin-tazobactam due to shortage.	Piperacillin-tazobactam	ADR	Vancomycin-resistant enterococci rates were similar. There was a 47% decrease in *Clostridium difficile* infections (p<0.001). This was associated with changed usage rates of particular antimicrobials.
Meloni, 2017 [49]	Nigeria	Cross-sectional study using samples and patient records of patients with HIV using antiretroviral therapy (ART). Treatment interruptions were caused by drug shortages.	Antiretrovirals	Other	Patients in the interrupted group had more drug resistance mutations to ART and less susceptibility to their ART than those in the uninterrupted group. Lower CD4+ count and higher viral load were reported in the interrupted group.
McLaughlin, 2017 [43]	USA	Retrospective cohort study using data from patient records. High dose oral valacyclovir (HDVA) was used to conserve IV acyclovir during a period of shortage.	IV Acyclovir	ADR	Six (40%) patients experienced at least one adverse drug reaction to HDVA. These reactions included thrombocytopenia, headache, nausea, and rash.
Hall, 2013 [29]	Canada	Survey of anaesthesiologists assessing reported impact on patient outcomes.	Drugs used in anaesthetics	Mortality ADR Drug error Hospitalisation Other	Changes in anaesthetic practice were reported as a result of shortages. Drug errors, complications, postponement of surgery, prolonged recovery and perioperative deaths were reported to be attributed to drug shortages. Improved outcomes were also reported such as less pruritis due to fentanyl shortage.
Roberts, 2012 [48]	USA	Retrospective cohort study using data from patient records. Alternative anaesthetic agents were used in non-cardiac ICU patients instead of propofol due to shortage.	Propofol	Hospitalisation Other	The alternative agents used instead of propofol resulted in an adjusted mean increase of duration mechanical ventilation of 7% (p = 0.35). The group which used alternative agents were more likely to receive continuous neuromuscular blocker therapy.
Romito, 2015 [34]	USA	Retrospective cohort study using data from patient records. Alternative agents were used for induction of anaesthesia due to propofol shortage.	Propofol	Mortality	Thirty-day and two-year mortality were similar between groups before and during the propofol shortage.
Price, 2013 [45]	USA	Retrospective cohort study using data from patient records. Ketamine was used instead of etomidate in patients requiring endotracheal intubation.	Etomidate	ADR Other	There were no concerning complications associated with replacement of etomidate with ketamine for endotracheal intubation. Similar haemodynamic changes and rates of adverse drug reactions were reported between groups.
Vail, 2017 [30]	USA	Retrospective cohort study using patient records from a medical database. Alternative vasopressors were used instead of norepinephrine in patients admitted with septic shock.	Norepinephrine	Mortality	Alternative vasopressors were used during a period of norepinephrine shortages, increasing absolute mortality rate 3.7% (p = 0.3).
Yan, 2017 [50]	Zambia	Mixed methods study where semi-structured interviews were conducted with healthcare providers at rural primary care facilities to gather insight into hypertension management. Other methods sought to review unrelated topics.	Antihypertensives	Other	Drug availability influenced prescribing of antihypertensives. Inferior treatments were commonly used because they were the only medications available.
Shah, 2015 [47]	UK	Mixed methods study. Retrospective cohort study using data from patient records, interviews with medical staff and patients.	Preservative-free glaucoma medicines	Hospitalisation	Patients with supply problems went to the emergency department to obtain their medication, some on multiple occasions.
Malone, 2016 [44]	UK	Prospective cohort study. Syntometrine (500 mcg ergometrine with 5 units oxytocin) was used as a uterotonic for non-emergent caesarean section due to shortage of IV oxytocin.	IV Oxytocin	ADR Other	Peri-operative outcomes such as blood transfusions, haemoglobin, and blood loss were similar between groups. Significantly more intra-operative antiemetics were given (p<0.001).
Ladha, 2015 [51]	USA	Retrospective cohort study using patient records. Alternative vasopressors were used due to a shortage of pharmacy-prepared ephedrine syringes.	Pharmacy-prepared ephedrine syringes	Other	Despite changes in vasopressor usage and lack of treatment guidelines, patient haemodynamics remained comparable.
Lukmanji, 2017 [22]	Canada	Mixed methods longitudinal cohort study using open-ended surveys of patients and patient records to study the impact of a clobazam shortage on patients with epilepsy.	Clobazam	ADR Other	Some patients reported increased side effects and increased seizures as a result of the shortage. Others reported they were not impacted.
Goldblatt, 2011 [52]	Australia	Prospective cohort study reporting outcomes when patients on imiglucerase as maintenance treatment for Gaucher’s disease were withdrawn from therapy due to shortage.	Imiglucerase	Other	Upon withdrawal of imiglucerase due to shortage 22/24 patients had no significant clinical complications. Eight patients complained of returning symptoms, two patients had to be recommenced on treatment.
Cho, 2016 [35]	USA	Retrospective cohort study using data from patient records. Courses of nimodipine for acute subarachnoid haemorrhage reduced from 21 days to 14 days due to shortage.	Nimodipine	Mortality ADR Hospitalisation Other	The shortened course of nimodipine was not associated with mortality, ADRs or LOS. Neurological outcomes and mechanical ventilation duration were also similar.
Blaine, 2016 [16]	USA	Retrospective cohort study using data from patient records. Tranexamic acid (TXA) was used instead of epsilon-aminocaproic acid (εACA) in cardiac surgery patients.	Epsilon-aminocaproic acid (εACA)	ADR Other	Substitution of εACA with TXA resulted in equivalent postoperative bleeding and red cell transfusions, patients receiving εACA were more likely to require supplemental haemostatic agents.

ADR = Adverse drug reaction LOS = Length of stay (in hospital)

Adverse drug reactions (ADRs) were reported in 24 studies. Of these, 14 reported increased ADRs [7, 13, 22, 22–26, 28, 33, 37, 38, 40, 43, 44], six reported equivalent incidences of ADRs [8, 31, 32, 35, 41, 45], and one reported decreased ADRs due to the shortages [16]. Three studies reported multiple impacts on ADRs. As above, the study by Abdelrahman et al. [36] reported both increased and equivalent ADRs, as it gathered physicians’ perspectives and there was a proportion of respondents who agreed, disagreed or were neutral with the statements in the survey. Hall et al. [29] reported both increased and decreased ADRs, where anaesthetic shortages were reported to increase post-operative complications, and fentanyl removal from epidurals resulted in less pruritis. Mendez et al. [39] reported both equivalent and decreased ADRs where piperacillin-tazobactam shortage did not affect the rate of vancomycin-resistant enterococci infections, the rate of *Clostridium difficile* infection decreased.

Drug errors were reported in nine studies. Eight of these reported an increase in drug errors [13, 19, 23, 24, 26, 29, 42, 46]. The study by Abdelrahman et al. [36] reported both increased and equivalent drug errors because it reported physicians’ perspectives.

Changes in hospitalisation status were reported in 11 studies. Of these seven studies reported increased hospitalisation [5, 13, 28, 29, 33, 37, 47], three reported equivalent hospitalisation [31, 35, 48], and one reported decreased hospitalisation [8].

A range of other clinical outcomes were also measured and reported in studies including drug-resistant mutations [49], use of inferior treatments [50, 51], rationing of medications [35, 52], quality of patient care [12, 13, 23], mechanical ventilation [48], and seizure frequency [22]. These can be seen in Table 2.

### Humanistic outcomes

Humanistic outcomes due to shortages were reported in eight studies (Table 3). Patient complaints were reported in three studies [5, 13, 29] and increased travel time was reported in four studies [13, 24, 26, 47]. Other outcomes reported included patients being frustrated, angry and feeling like a burden to themselves and caregivers. These can be seen in Table 3. One study by Lukmanji et al. [22] used a quantitative quality of life (QOL) tool which found no statistical difference between participants QOL pre- and during-shortage. The same study also collected qualitative quotes from participants,—“When I first found out about the shortage I panicked and I was immediately worried that I was going to run out of pills and thought far ahead–I have a problem with catastrophic thinking. I thought far ahead about if I ran out of [Frisium] and if I have a seizure and worried about losing my license and house and then losing AISH [financial support]. When it got to that point I thought about killing myself. The anxiety got that bad” demonstrating that there were some impacts on patients’ humanistic outcomes not captured via the QOL tool.

**Table 3:** Humanistic outcomes of drug shortages on patients.

First author and year	Location	Study Description	Shortage	Type of outcome	Description of outcome
McLaughlin, 2013 [13]	USA	Survey of pharmacy directors assessing the effects of patient care caused by drug shortages.	General medicines	Complaints Travel	Patient complaints were reported by 38% of respondents. Patients having to be transferred to another facility which had the shortage medication was reported by 12.3%.
Walker, 2017 [5]	Fiji	Semi-structured interviews of key stakeholders in the Fijian medicine supply chain.	General medicines	Complaints	Patients were reported to be angry with pharmacists due to shortages.
Goldsack, 2014 [24]	USA	Survey of pharmacists on the impact of shortages of injectable oncology drugs on patient care.	Oncology medicines	Travel	Pharmacists reported that patients were often referred to and from other facilities as a result of drug shortages.
McBride, 2013 [26]	USA	Survey of oncology pharmacists on the effect of oncology drug shortages on cancer care.	Oncology medicines	Travel	Some institutions reported sending medicines to a patient at another institution or accepting patients from other institutions that could not obtain required medicines.
Hall, 2013 [29]	Canada	Survey of anaesthesiologists assessing reported impact on patient outcomes.	Drugs used in anaesthetics	Complaints	Patient complaints due to drug shortages were reported by 2.3% of anaesthesiologists.
Perumal-Pillay, 2017 [20]	South Africa	Focus groups of parents and guardians of children gathering opinions and perceptions on availability of children’s medications.	Paediatric medicines	Concerns	Participants were concerned if the generic medications were going to have the same effect as the ‘proper’ medication.
Shah, 2015 [47]	UK	Mixed methods study. Retrospective cohort study using data from patient records, interviews with medical staff and patients.	Preservative-free glaucoma medicines	Concerns Travel	Patients were reported to be confused, distressed, frustrated and angry about the situation and anxious about reactions to alternative drops. Some patients reported travelling long distances to obtain supplies.
Lukmanji, 2017 [22]	Canada	Mixed methods longitudinal cohort study using open-ended surveys of patients and patient records to study the impact of a clobazam shortage on patients with epilepsy.	Clobazam	Quality of life Concerns Burden	There were no statistically significant differences in participants’ pre- and during shortage responses on epilepsy-related quality of life distress and fear of having a seizure using the QOLIE-10-P tool. Qualitative analysis revealed themes such as burden on patients and caregivers, physical and psychological impacts of the shortage. Participants emphasised the increased financial and time burden that the clobazam shortage imposed on patients and their caregivers.

## Discussion

The results of this review demonstrate that medication shortages are a complex, global phenomenon, which affects patients' economic, clinical, and humanistic outcomes. While drug shortages have been reported to be a global issue which are reported by 99% of pharmacists each year [19], there were only 40 studies gained from our comprehensive search strategy which reported patient outcomes that met inclusion criteria for review. The reasons for such underreporting may be due to the problem being so ubiquitous that no one has ever really questioned it, or that drug shortages are a new phenomenon which has not yet been fully explored, or that clinician time is spent dealing with workarounds, and time for research, audits, documentation and follow-up is not available. Even though research evidence of patient consequence may be an underreported phenomenon, this review highlighted that regardless of the medicine that was in short supply, the majority of patient outcomes resulting from the shortage were disadvantageous to patients’ clinical, economic and humanistic outcomes.

With respect to economic outcomes, these were only reported in five studies. This could be attributed to lack of economic data generated as the majority of studies focused on clinical outcomes and used retrospective chart analysis for information. Economically, drug shortages in all reported instances increased OOP costs for patients. These increased costs were attributed to factors such as switching brand of the same medicine, switching to an alternative medicine, and expenses such as fuel for travelling further distances to acquire medicines. Patient OOP expenses were the only economic outcome reported, perhaps this may be due to health research being biased towards clinical outcomes or that it is difficult to capture the actual economic cost of drug shortages. No studies reported other economic outcomes such as productivity costs (e.g. time off work) or cost-utility, demonstrating that there may be a lack of sophistication in analysing the actual economic impact that shortages have on patients [53].

It should be noted that other economic considerations were reported in some studies such as institutional costs. One institution reported having to pay 300–500% more for shortage medicines [46], another reported having to pay up to 1704% more for an alternative agent [40]. Furthermore, studies reported having to have dedicated staff to manage shortages [30]. As many of these studies were from the USA, it is unknown if or how these costs would be passed onto the patients directly or their insurance companies and affect premiums and as these other economic considerations were not reported to affect patients directly, we can only theorise their impacts. Furthermore, different countries have different healthcare systems, where the costs of access to medicines during times of shortage may vary.

Clinical outcomes were reported in the majority of studies. However, these were generally retrospective reviews of data from patient records. Data gathered via this medium included specific patient outcomes related to the treatment, such as rates of infection or seizures. Other outcomes such as adverse drug reactions and drug errors were also often gathered via this retrospective audit of notes. Utilising this modality may have its limitations, as clinical documentation may not always be complete [54]. For example, studies which reported only on adverse events did not give a full depiction as to whether the alternative treatments were beneficial in treating the primary condition [38, 39]. The other method utilised to report the clinical outcomes of drug shortages were self-report mainly from clinicians via survey or semi-structured interviews. These data collection methods may also lead to a problematic interpretation of the actual impact of the medication shortages on patient outcomes, as they may generate recall bias, particularly if participants were surveyed some-time after the shortage. Furthermore, survey questions may be leading, inflating the perceived outcomes of the shortage, also, these methods are only reporting perceptions of the impact of the shortage on the patient from the lens of the health professional and not from the patients themselves.

Rates of adverse events were reported in 20 studies. The majority of reports indicated an increase in adverse events such as increased toxicity of the alternative treatment. Interestingly, two studies reporting on the impact of piperacillin-tazobactam shortages on secondary *Clostridium difficile* infection and had conflicting results. One study reported an increased rate of *Clostridium difficile* infection [38], whereas the other, a decrease [39]. The changes in infection rate and adverse events may be attributed to the alternative therapy used, rather than the shortage itself. Thus, in order to use data from shortages, comprehensive clinical documentation is needed to guide future research and treatment protocols, particularly if improved responses to alternative treatments are reported. Interestingly, many of the studies reviewed were regarding drug shortages of antimicrobials or oncology medicines. These shortages may be more likely to be studied, due to the perceived importance placed on these medicines. However due to the heterogenous reporting of patient outcomes across studies, few comparisons of results can be compared.

Drug shortages were also associated with increased medication errors. This was attributed to factors such as unfamiliarity with alternative agents. Pharmacy staff noted in a 2003 study reporting on 109 shortages at one institute that in 54% of shortages clinicians may be unfamiliar with the alternative product regarding its mechanism of action, adverse effects, or interactions [55].

Lack of medication availability causing death is the most severe consequence of drug shortages and mortality was reported in 18 studies. Some of these studies reported few deaths, whereas others could attribute hundreds. In a 5-year retrospective cohort study of 27,835 patients with septic shock during a norepinephrine shortage, alternative vasopressor use resulted in an increased mortality of 3.7% (p = 0.3) [30]. In contrast to this long-term comparative study, others directly gathered physician perspectives on the relationships between drug shortages and mortality. This was the case in the study by Abdelrahman et al. where approximately 1/3 of physicians stated that shortages caused death, 1/3 said they did not, and 1/3 responded neutrally on the survey tool [36]. These heterogeneous methods used to report the impact of shortages, again making it difficult to draw firm conclusions on true impacts, and more comprehensive studies comparing those receiving no drug, or alternative treatment are required to highlight the full clinical consequence of shortages.

In addition to clinical and economic outcomes, it has been stated that health care is more than just treating a condition, and humanistic outcomes such as quality of life is also an important measure of successful health care [56]. However, in our review, we found only ten studies which reported humanistic outcomes. These studies reported patients having difficulty accessing their medications, patients complaining to health professionals about drug shortages, patients having to be transferred to other facilities to find suitable health care, and patients being anxious and distressed. One study used a quantitative tool to measure QOL during a clobazam shortage and reported no significant difference [22]. However, within the same study, there were extracts from patient interviews which suggest otherwise. Previous literature has also reported that often QOL tools may not be sensitive enough to capture the true impact of an intervention on the patient and that other tools measuring humanistic outcomes may need to be developed [56]. It is doubtful that this review captured the entire range of humanistic impacts of drug shortages, however, those reported predominantly seem to be negatively affecting the patient. Further research investigating humanistic outcomes and the patients’ perspective of how drug shortages affect them is warranted in order to create holistic solutions to the shortage problem.

It has been commonly reported that drug shortages are a global problem. However, the majority of the studies included in this review were from developed nations such as the United States. The World Health Organisation reports that up to a third of the world’s population does not have access to medications, where this rate is even higher in low-to-middle income countries [57], yet these nations are not well represented in this review. This prompts the question as to whether the impacts of drug shortages may differ in these nations. Due to the majority of studies reporting outcomes of shortages in developed countries, the results found in this review may not be generalisable to developing countries.

Due to the dearth of research literature reporting patient outcomes due to medication shortages, as well as the lack of homogeneity in the tools used to report these outcomes, this scoping review provides a snapshot of some of the economic, clinical and humanistic outcomes that may result from medication shortages. However, it may not allow one to make any firm conclusions about their true impact on patient outcomes.

It should be noted that much of the data comes from study types of low research validity, as the majority of studies were retrospective cohort studies or surveys. While cohort studies are observations of real outcomes of shortages, they may lack rigour. Surveys of healthcare professionals were the other common research method utilised. As mentioned, these are subject to biases and only gather the perspectives of the clinicians and not the patients themselves who are experiencing the treatment changes due to the shortage. Only one study in this review, actually asked patients themselves about the impacts they faced as a result of their medication shortage [22]. Additionally, the researchers may have had preconceived ideas of what data to record and may not have reported the full impact of the shortage for each individual involved. We believe a recording method for shortages should be created in order to capture the entire clinical, economic, and humanistic picture of drug shortages for the future. Due to the nature and small numbers of these manuscripts, the risk of bias was not assessed in this review.

This scoping review therefore had both strengths and limitations. This is one of the first reviews that we are aware of attempting to synthesise the literature on the consequences of drug shortages specifically on patient outcomes. The systematic and comprehensive search strategy in many academic databases and the use of two authors applying the inclusion and exclusion criteria across all articles lead us to believe that we have captured most of the research data in this field. However, limiting the search to English only and not searching grey literature may mean that some studies (and accounts) have been missed. In addition, this review did not attempt to extract the root cause of these shortages or the strategies that have been put in place to overcome drug shortages when they occur. Lists of root causes and strategies can often be found on National Health System websites [6, 58]. Furthermore, it did not attempt to make any comparisons between different health systems, and as already stated, much of the data was from developed nations and studies reporting patient outcomes in other parts of the world were lacking.

Whilst it was difficult to combine outcomes from different study types, this review highlights the need for creation of guidelines or a tool to record the impacts of drug shortages for future in order to allow better comparison and reporting of meaningful outcomes. More structured data should be gathered during times of shortage, which in turn can be used to guide further research and treatment protocols. Particularly, the economic and humanistic impact of medication shortages needs to be addressed.

## Conclusion

The results of this review provide valuable insights into the impact drug shortages have on patient outcomes. The majority of studies reported medication shortages resulted in negative patient clinical, economic and humanistic outcomes.

## Supporting information

S1 File PRISMA checklist. (DOC)

S2 File Sample search strategy. (DOCX)

## References

[1] World Health Organisation. Medicines and health products. 2018 [cited 7 April 2018] In: World Health Organisation [Internet] 2018. Available from: http://www.who.int/healthsystems/topics/medicines/en/

[2] Hedman L. Global approaches to addressing shortages of essential medicines in health systems. WHO Drug Information. 2016;30(2):180–5.

[3] Holcombe B, Mattox TW, Plogsted S. Drug Shortages: Effect on Parenteral Nutrition Therapy. Nutr Clin Pract. 2018;33(1):53–61. 10.1002/ncp.10052 29365360

[4] Mori J, Hasui K, Tanimoto T, Matsumura T, Kami M. Drug Shortages After the Eastern Japan Earthquake: Experiences in a Tertiary Referral Center. Drug Inf J. 2012;46(5):607–10.

[5] Walker J, Chaar BB, Vera N, Pillai AS, Lim JS, Bero L, et al Medicine shortages in Fiji: A qualitative exploration of stakeholders' views. PLoS ONE. 2017;12(6):e0178429 10.1371/journal.pone.0178429 28582409 PMC5459560

[6] U.S. Food and Drug Administration. Drug Shortages Infographic. 2017 1 8 [cited 7 April 2018] In: U.S. Food and Drug Administration [Internet] 2016. Available from: https://www.fda.gov/downloads/Drugs/DrugSafety/DrugShortages/UCM441583.pdF

[7] Tan YX, Moles RJ, Chaar BB. Medicine shortages in Australia: causes, impact and management strategies in the community setting. Int J Clin Pharm. 2016;38(5):1133–41. 10.1007/s11096-016-0342-1 27383246

[8] Thoma BN, Li J, McDaniel CM, Wordell CJ, Cavarocchi N, Pizzi LT. Clinical and economic impact of substituting dexmedetomidine for propofol due to a US drug shortage: Examination of coronary artery bypass graft patients at an urban medical centre. PharmacoEconomics. 2014;32(2):149–57. 10.1007/s40273-013-0116-8 24254138

[9] U.S Food and Drug Administration. Current and Resolved Drug Shortages and Discontinuations Reported to FDA 2018 April 7 [cited 7 April 2018]. In: U.S. Food and Drug Administration [Internet] 2018. Available from: https://www.accessdata.fda.gov/scripts/drugshortages/default.cfm

[10] American Society of Health-System Pharmacists. Drug Shortages Statistics. 2017 [cited 7 April 2018] In: ASHP [Internet] 2018. Available from: https://www.ashp.org/Drug-Shortages/Shortage-Resources/Drug-Shortages-Statistics

[11] The Society of Hospital Pharmacists of Australia. Medication shortages in Australia. 2017 [cited 11 April 2018] In: SHPA [Internet] 2018. Available from: https://www.shpa.org.au/sites/default/files/uploaded-content/website-content/shpa_medicines_shortages_in_australia_report_june_2017.pdf

[12] Al Ruthia YS, Al Kofide H, Al Ajmi R, Balkhi B, Alghamdi A, Al Nasser A, et al Drug shortages in large hospitals in Riyadh: A cross-sectional study. Ann Saudi Med. 2017;37(5):375–85. 10.5144/0256-4947.2017.375 28988252 PMC6074191

[13] McLaughlin M, Kotis D, Thomson K, Harrison M, Fennessy G, Postelnick M, et al Effects on patient care caused by drug shortages: A survey. J Manag Care Pharm. 2013;19(9):783–8. 10.18553/jmcp.2013.19.9.783 24156647 PMC10437927

[14] Souliotis K, Papageorgiou M, Politi A, Ioakeimidis D, Sidiropoulos P. Barriers to accessing biologic treatment for rheumatoid arthritis in Greece: The unseen impact of the fiscal crisis—The Health Outcomes Patient Environment (HOPE) study. Rheumatol Int. 2014;34(1):25–33. 10.1007/s00296-013-2866-1 24057144

[15] Rider AE, Templet DJ, Daley MJ, Shuman C, Smith LV. Clinical dilemmas and a review of strategies to manage drug shortages. J Pharm Pract. 2013;26(3):183–91. 10.1177/0897190013482332 23553544

[16] Blaine KP, Press C, Lau K, Sliwa J, Rao VK, Hill C. Comparative effectiveness of epsilon-aminocaproic acid and tranexamic acid on postoperative bleeding following cardiac surgery during a national medication shortage. J Clin Anesth. 2016;35:516–23. 10.1016/j.jclinane.2016.08.037 27871586

[17] Moher D, Liberati A, Tetzlaff J, Altman DG, The PG. Preferred Reporting Items for Systematic Reviews and Meta-Analyses: The PRISMA Statement. PLoS Med. 2009;6(7):e1000097 10.1371/journal.pmed.1000097 19621072 PMC2707599

[18] Sollaci LB, Pereira MG. The introduction, methods, results, and discussion (IMRAD) structure: a fifty-year survey. J Med Libr Assoc. 2004;92(3):364–71. 15243643 PMC442179

[19] Pauwels K, Simoens S, Casteels M, Huys I. Insights into European Drug Shortages: A survey of hospital pharmacists. PLoS ONE. 2015;10 (3):e0119322 10.1371/journal.pone.0119322 25775406 PMC4361582

[20] Perumal-Pillay VA, Suleman F. Parents' and guardians' perceptions on availability and pricing of medicines and healthcare for children in eThekwini, South Africa—a qualitative study. BMC Health Serv Res. 2017;17(1):417 10.1186/s12913-017-2385-y 28629443 PMC5477259

[21] Hayes MS, Ward MA, Slabaugh SL, Xu Y. Lessons from the leucovorin shortages between 2009 and 2012 in a medicare advantage population: Where do we go from here? Am Health Drug Benefits. 2014;7(5):264–70. 25237422 PMC4163778

[22] Lukmanji S, Sauro KM, Josephson CB, Altura KC, Wiebe S, Jette N. A longitudinal cohort study on the impact of the clobazam shortage on patients with epilepsy. Epilepsia. 2018;59(2):468–78. 10.1111/epi.13974 29218701

[23] Baumer AM, Clark AM, Witmer DR, Geize SB, Vermeulen LC, Deffenbaugh JH. National survey of the impact of drug shortages in acute care hospitals. Am J Health Syst Pharm. 2004;61(19):2015–22. 10.1093/ajhp/61.19.2015 15509124

[24] Goldsack JC, Reilly C, Bush C, McElligott S, Bristol MN, Motanya UN, et al Impact of shortages of injectable oncology drugs on patient care. Am J Health Syst Pharm. 2014;71(7):571–8. 10.2146/ajhp130569 24644117

[25] Kehl KL, Gray SW, Kim B, Kahn KL, Haggstrom D, Roudier M, et al Oncologists' experiences with drug shortages. J Oncol Pract. 2015;11(2):e154–e62. 10.1200/JOP.2014.000380 25549653 PMC4371121

[26] McBride A, Holle LM, Westendorf C, Sidebottom M, Griffith N, Muller RJ, et al National survey on the effect of oncology drug shortages on cancer care. Am J Health Syst Pharm. 2013;70(7):609–17. 10.2146/ajhp120563 23515514

[27] Berger JL, Smith A, Zorn KK, Sukumvanich P, Olawaiye AB, Kelley J, et al Outcomes analysis of an alternative formulation of PEGylated liposomal doxorubicin in recurrent epithelial ovarian carcinoma during the drug shortage era. Onco Targets Ther. 2014;7:1409–13. 10.2147/OTT.S62881 25143745 PMC4133030

[28] Gundlapalli AV, Beekmann SE, Graham DR, Polgreen PM. Perspectives and concerns regarding antimicrobial agent shortages among infectious disease specialists. Diagn Microbiol Infect Dis. 2013;75(3):256–9. 10.1016/j.diagmicrobio.2012.11.020 23305775 PMC5815827

[29] Hall R, Bryson GL, Flowerdew G, Neilipovitz D, Grabowski-Comeau A, Turgeon AF. Drug shortages in Canadian anesthesia: A national survey. Can J Anaesth. 2013;60(6):539–51. 10.1007/s12630-013-9920-z 23546924

[30] Vail E, Gershengorn HB, Hua M, Walkey AJ, Rubenfeld G, Wunsch H. Association between US norepinephrine shortage and mortality among patients with septic shock. JAMA. 2017;317(14):1433–42. 10.1001/jama.2017.2841 28322415

[31] Nickel RS, Keller F, Bergsagel J, Cooper T, Daves M, Sabnis H, et al Mitoxantrone as a substitute for daunorubicin during induction in newly diagnosed lymphoblastic leukemia and lymphoma. Pediatr Blood Cancer. 2014;61(5):810–4. 10.1002/pbc.24892 24357218 PMC4317248

[32] Trifilio S, Zhou Z, Mehta J, Czerniak C, Pi J, Greenberg D, et al Idarubicin appears equivalent to dose-intense daunorubicin for remission induction in patients with acute myeloid leukemia. Leuk Res. 2013;37(8):868–71. 10.1016/j.leukres.2013.04.009 23726414

[33] Dilworth TJ, Ibrahim OM, Mercier RC. Impact of an intravenous trimethoprim/sulfamethoxazole shortage on treatment outcomes among HIV-infected patients with Pneumocystis jirovecii pneumonia. J Manag Care Pharm. 2014;20(12):1246–54. 10.18553/jmcp.2014.20.12.1246 PMC10441019 25443518

[34] Romito B, Stone J, Ning N, Yin C, Llano EM, Liu J, et al How drug shortages affect clinical care: The case of the surgical anesthetic propofol. Hosp Pharm. 2015;50(9):798–805. 10.1310/hpj5009-798 26912921 PMC4750830

[35] Cho S, Bales J, Tran TK, Korab G, Khandelwal N, Joffe AM. Effects of 14 Versus 21 Days of Nimodipine Therapy on Neurological Outcomes in Aneurysmal Subarachnoid Hemorrhage Patients. Ann Pharmacother. 2016;50(9):718–24. 10.1177/1060028016653138 27273676

[36] Abdelrahman AA, Saad AA, Sabry NA, Farid SF. Perceptions of Egyptian physicians about drug shortage during political disturbances: Survey in Greater Cairo. Bulletin of Faculty of Pharmacy, Cairo University. 2016;54(2):191–6.

[37] McLaughlin MM, Skoglund E, Pentoney Z, Scheetz MH. Developing a Method for Reporting Patient Harm Due to Antimicrobial Shortages. Infect Dis Ther. 2014;3(2):349–55. 10.1007/s40121-014-0040-z 25234281 PMC4269628

[38] Gross AE, Johannes RS, Gupta V, Tabak YP, Srinivasan A, Bleasdale SC. The Effect of a Piperacillin/Tazobactam Shortage on Antimicrobial Prescribing and Clostridium difficile Risk in 88 US Medical Centers. Clin Infect Dis. 2017;65(4):613–8. 10.1093/cid/cix379 28444166 PMC11320714

[39] Mendez MN, Gibbs L, Jacobs RA, McCulloch CE, Winston L, Guglielmo BJ. Impact of a piperacillin-tazobactam shortage on antimicrobial prescribing and the rate of vancomycin-resistant Enterococci and Clostridium difficile infections. Pharmacotherapy. 2006;26(1):61–7. 16509027 10.1592/phco.2006.26.1.61

[40] Becker DJ, Talwar S, Levy BP, Thorn M, Roitman J, Blum RH, et al Impact of oncology drug shortages on patient therapy: Unplanned treatment changes. J Oncol Pract. 2013;9(4):e122–e8. 10.1200/JOP.2012.000799 23942928 PMC3710178

[41] Duan F, Wang EQ, Lam MG, Abdelmaksoud MH, Louie JD, Hwang GL, et al Superselective Chemoembolization of HCC: Comparison of Short-term Safety and Efficacy between Drug-eluting LC Beads, QuadraSpheres, and Conventional Ethiodized Oil Emulsion. Radiology. 2016;278(2):612–21. 10.1148/radiol.2015141417 26334787

[42] Salazar EG, Bernhardt MB, Li Y, Aplenc R, Adamson PC. The impact of chemotherapy shortages on COG and local clinical trials: A report from the children's oncology group. Pediatr Blood Cancer. 2015;62(6):940–4. 10.1002/pbc.25445 25704486 PMC4670038

[43] McLaughlin MM, Sutton SH, Jensen AO, Esterly JS. Use of High-Dose Oral Valacyclovir During an Intravenous Acyclovir Shortage: A Retrospective Analysis of Tolerability and Drug Shortage Management. Infect Dis Ther. 2017;6(2):259–64. 10.1007/s40121-017-0157-y 28417331 PMC5446370

[44] Malone C, Acheson JR, Hinds JD, McComiskey MH. Uterotonics for non-emergent caesarean section: Protocol change during UK-licensed drug shortage. Ulster Med J. 2016;85(3):174–7. 27698519 PMC5031104

[45] Price B, Arthur AO, Brunko M, Frantz P, Dickson JO, Judge T, et al Hemodynamic consequences of ketamine vs etomidate for endotracheal intubation in the air medical setting. Am J Emerg Med. 2013;31(7):1124–32. 10.1016/j.ajem.2013.03.041 23702065

[46] Caulder C, Mehta B, Bookstaver P, Sims L, Stevenson B. Impact of drug shortages on health system pharmacies in the Southeastern United States. Hosp Pharm. 2015;50(4):279–86. 10.1310/hpj5004-279 26448658 PMC4589883

[47] Shah S, Theodossiades J, Chapman K, Murdoch I. Impact of supply problems of preservative-free glaucoma medications on patients and hospital staff. Ophthalmic Physiol Opt. 2015;35(2):236–41. 10.1111/opo.12180 25761582

[48] Roberts R, Ruthazer R, Chi A, Grover A, Newman M, Bhat S, et al Impact of a national propofol shortage on duration of mechanical ventilation at an academic medical center. Crit Care Med. 2012;40(2):406–11. 10.1097/CCM.0b013e31822f0af5 21926579

[49] Meloni ST, Chaplin B, Idoko J, Agbaji O, Akanmu S, Imade G, et al Drug resistance patterns following pharmacy stock shortage in Nigerian Antiretroviral Treatment Program. AIDS Res Ther. 2017;14 (1) (no pagination)(58). 10.1186/s12981-017-0141-3 29029637 PMC5640939

[50] Yan LD, Chirwa C, Chi BH, Bosomprah S, Sindano N, Mwanza M, et al Hypertension management in rural primary care facilities in Zambia: a mixed methods study. BMC Health Serv Res. 2017;17(1):111 10.1186/s12913-017-2063-0 28158981 PMC5292001

[51] Ladha KS, Nanji KC, Pierce E, Poon KT, Hyder JA. The impact of a shortage of pharmacy-prepared ephedrine syringes on intraoperative medication use. Anesth Analg. 2015;121(2):404–9. 10.1213/ANE.0000000000000809 26076388

[52] Goldblatt J, Fletcher JM, McGill J, Szer J, Wilson M. Enzyme replacement therapy "drug holiday": Results from an unexpected shortage of an orphan drug supply in Australia. Blood Cells Mol Dis. 2011;46(1):107–10. 10.1016/j.bcmd.2010.05.002 20684886

[53] Salkeld G, Davey P, Arnolda G. A critical review of health-related economic evaluations in Australia: implications for health policy. Health Policy. 1995;31(2):111–25. 10141252 10.1016/0168-8510(94)00672-5

[54] Pirkle CM, Dumont A, Zunzunegui M-V. Medical recordkeeping, essential but overlooked aspect of quality of care in resource-limited settings. Int J Qual Health Care. 2012;24(6):564–7. 10.1093/intqhc/mzs034 22798693

[55] Fox ER, Tyler LS. Managing drug shortages: seven years’ experience at one health system. Am J Health Syst Pharm. 2003;60(3):245 10.1093/ajhp/60.3.245 12613233

[56] Mohammed M, Moles R, F Chen T. The Impact of Pharmaceutical Care Interventions on Health-Related Quality-of-Life Outcomes: A Systematic Review and Meta-analysis. Ann Pharmacother. 2016;50(10):862–81. 10.1177/1060028016656016 27363846

[57] Duong M, Moles RJ, Chaar B, Chen TF. Essential Medicines in a High Income Country: Essential to Whom? PLOS ONE. 2015;10(12):e0143654 10.1371/journal.pone.0143654 26650544 PMC4674059

[58] NHS Scotland. Best Practice Standards for Managing Medicines Shortages in Secondary Care in Scotland. 2017 [Cited 20 March 2019] In: NHS Scotland [Internet] 2019. Available from: https://www.rpharms.com/Portals/0/RPS%20document%20library/Open%20access/News/best-practice-standards-for-managing-medicine-shortages-in-secondary-care-in-scotland-final-v1.pdf?ver=2017-03-20-150944-587

