# Supplementary material for: The impacts of medication shortages on patient outcomes: A scoping review
Source: PLoS One. 2019 May 3;14(5):e0215837. doi: 10.1371/journal.pone.0215837 (PMC6499468; doi:10.1371/journal.pone.0215837)
Supplement: S2 File — (DOCX) [file pone.0215837.s002.docx]

**S2 File – Sample search strategy**

Database: Embase Classic <1947 to 1973>, Embase <1974 to 2018 June 12>

Search Strategy:

--------------------------------------------------------------------------------

1 medication* shortage*.mp.

2 medicine* shortage*.mp.

3 drug* shortage*.mp.

4 patient*.mp.

5 impact*.mp.

6 outcome*.mp.

7 1 or 2 or 3

8 5 or 6

9 4 and 7 and 8

10 remove duplicates from 9

11 limit 10 to english language

12 limit 11 to human

***************************
